# Spectroscopic observation of two-center three-electron bonded (hemi-bonded) structures of (H_2_S)_*n*_^+^ clusters in the gas phase†
†Electronic supplementary information (ESI) available: Calculated relative energies of stable isomers of (H_2_S)_*n*_^+^ (*n* = 3–6), calculated harmonic frequencies of (H_2_S)_4_^+^ at the different calculation levels, comparison between the observed and simulated spectra of (H_2_S)_*n*_^+^ (*n* = 3–6). See DOI: 10.1039/c6sc05361k



**DOI:** 10.1039/c6sc05361k

**Published:** 2017-01-11

**Authors:** Dandan Wang, Asuka Fujii

**Affiliations:** a Department of Chemistry , Graduate School of Science , Tohoku University , Sendai 980-8578 , Japan . Email: asukafujii@m.tohoku.ac.jp

## Abstract

A two-center three-electron 2c–3e bond (hemi-bond) is a non-classical chemical bond, and its existence has been supposed in radical cation clusters with lone pairs. Though the nature of the hemi-bond and its role in the reactivity of radical cations have attracted great interest, spectroscopic observations of hemi-bonded structures have been very scarce. In the present study, the presence of a stable hemi-bonded core (H_2_S∴SH_2_)^+^ in (H_2_S)_*n*_^+^ (*n* = 3–6) in the gas phase is demonstrated by infrared spectroscopy combined with quantum chemical calculations. The spectral features of the free SH stretch of the ion core show that the hemi-bond motif of the ion core is maintained up to the completion of the first H-bonded solvation shell. All of the observed spectra are well reproduced by the minimum energy hemi-bonded isomers, and no sign of the proton-transferred ion core type H_3_S^+^–SH, which is estimated to have a much higher energy, is found. Spin density calculations show that the excess charge is almost equally delocalized over the two H_2_S molecules in the cluster for *n* = 3 to 6. This also indicates the hemi-bond nature of the (H_2_S∴SH_2_)^+^ ion core and the small impact of the formation of a solvation shell on the ion core.

## Abstract 2

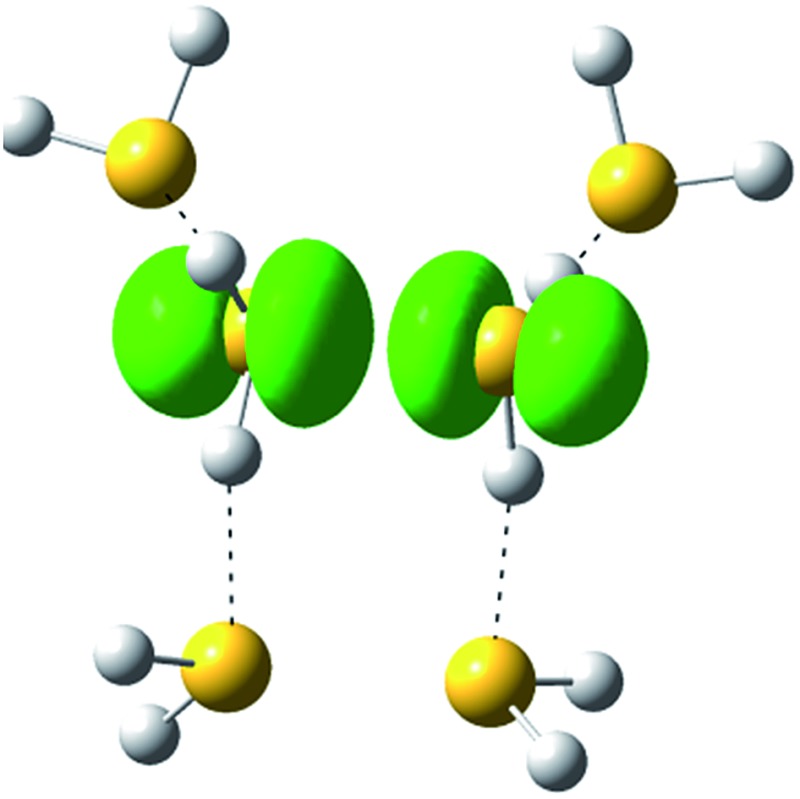
The presence of a two-center three-electron (2c–3e) bonded (hemi-bonded) ion core in the (H_2_S)_*n*_^+^ cluster is revealed by infrared spectroscopy combined with *ab initio* calculations. The stability of the hemi-bonded ion core to solvation is also proved.

## Introduction

When lone pair orbitals of a neutral molecule and its radical cation overlap and molecular orbitals are formed, the bonding σ orbital is doubly occupied while the antibonding σ* orbital is singly occupied. The formal bond order of this interaction is 1/2, indicating the formation of a stable bond between the two molecules.^1–3^ Such a bond is called a two-center three-electron (2c–3e) bond or hemi-bond. Since the first characterization of the hemi-bond by Linus Pauling in the early 1930s,^4^ the hemi-bonds of radical cations have attracted strong interest in biological, atmospheric, and radiation chemistry, and the role of the hemi-bond has been discussed to understand the reactivity of radical cations.^5,6^

**Fig. 1:**
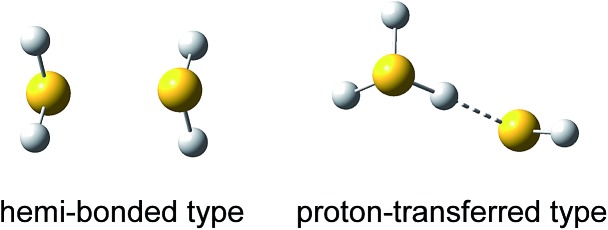
Two possible structural motifs of (H_2_S)_2_^+^.

One of the simple model systems to investigate hemi-bonded radical cations is (H_2_O)_*n*_^+^. In spite of many theoretical studies so far, however, hemi-bonded structures of (H_2_O)_*n*_^+^ have not yet been experimentally observed because of the strong competition of the formation of the proton-transferred H_3_O^+^–OH ion core type.^7–10^ On the other hand, (H_2_S)_*n*_^+^ seems to be more feasible for a hemi-bond study. Several theoretical studies of (H_2_S)_2_^+^ have predicted that the hemi-bonded structure (H_2_S∴SH_2_)^+^ is much more stable than the proton-transferred structure H_3_S^+^–SH (see Fig. 1) by *ca.* 50–100 kJ mol^–1^, depending on the level of theory.^5,11–13^ This preference for the hemi-bonded type structure in (H_2_S)_2_^+^ is in contrast to its analogues along period 2. Furthermore, an energy decomposition scheme has been applied to (H_2_S∴SH_2_)^+^, and it has shown that nearly 60% of the sulfur–sulfur bond is provided by the three-electron bond but that electrostatic attraction also makes a large contribution (∼40%) to the bond.^14^ This energy decomposition scheme neglects electron correlation. Therefore, we should note that dispersion may also play an important role in such a system.^15^

Despite these theoretical studies which predict the energetic superiority of the hemi-bonded structure over the proton-transferred structure, very few experimental studies have been reported on (H_2_S)_*n*_^+^ and their analogues. Until now, the sulfur–sulfur hemi-bond has been characterized only by EPR and UV-vis (transient) absorption.^16–18^ However, it is very difficult to directly extract structural information from these types of experiments. Spectroscopic evidence in the gas phase has been highly requested to examine the theoretical predictions of the sulfur–sulfur hemi-bond.

Recently, the weakening of a hemi-bond through the delocalization of the spin density beyond two nuclei centers has been suggested.^17,19^ This phenomenon implies the potential influence of hydrogen bonds (H-bonds) on the hemi-bond since charge transfer by orbital overlap frequently occurs with the formation of an H-bond.^20^ The influence of H-bonds (solvation) on the hemi-bonded ion core should be explored.

In this study, to address the issues proposed above, we report an infrared (IR) spectroscopic study of (H_2_S)_*n*_^+^ (*n* = 3–6) in the gas phase. The presence of the (H_2_S∴SH_2_)^+^ hemi-bonded ion core is revealed for all observed sizes, and the evolution of the solvation structure is characterized. The experimental observations are consistent with the favorability of the hemi-bonded ion core over the proton-transferred ion core in (H_2_S)_*n*_^+^ as predicted by recent theoretical calculations.^12^

## Results and discussion

**Fig. 2:**
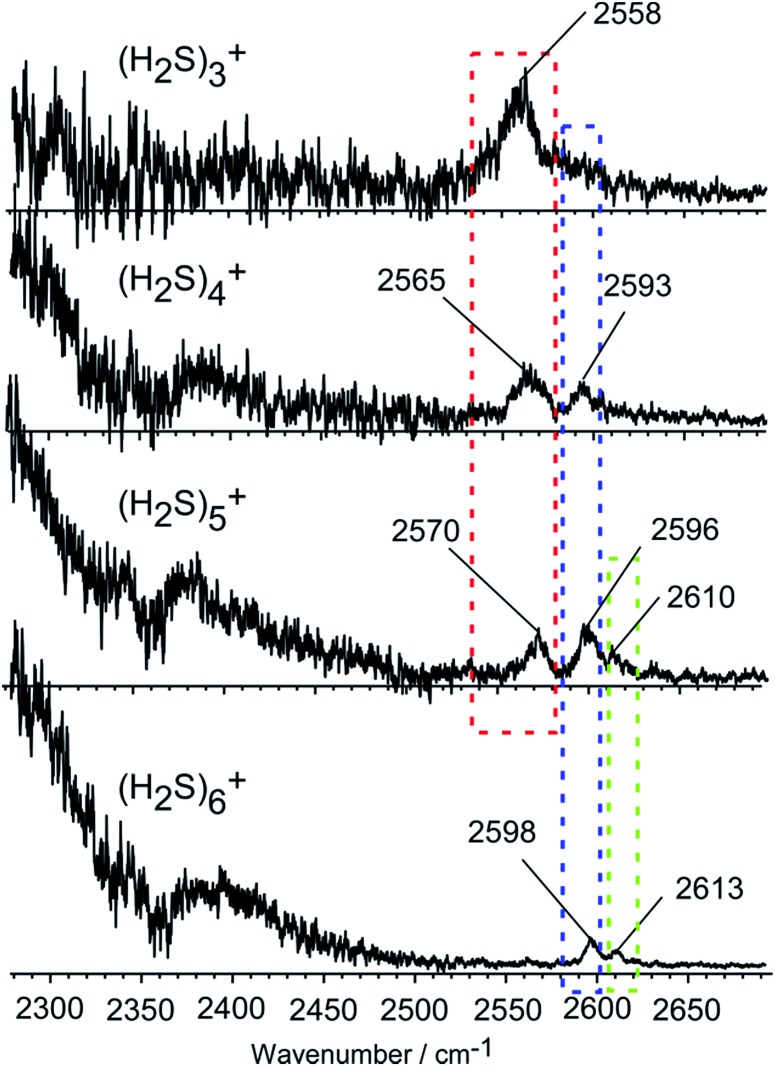
Observed IR spectra of (H_2_S)_*n*_^+^ (*n* = 3–6). The bands are categorized into three types by colored dotted blocks (see text). The bump at ∼2400 cm^–1^, present throughout all cluster sizes, is caused by the depletion at 2360 cm^–1^ due to the strong IR absorption by atmospheric CO_2_.

Fig. 2 shows the observed IR spectra of (H_2_S)_*n*_^+^ (*n* = 3–6) in the SH stretch region. The bands higher than 2550 cm^–1^ are attributed to free SH stretches, and they are categorized into three different types of vibration, as indicated by colored dotted blocks in the figure. Because of the ionization, the SH bonds in the ion core should be somewhat weakened, so their stretch frequency is expected to be lower than that of neutral H_2_S. Therefore, the lowest frequency band at around 2560 cm^–1^ in each size (the band in the red dotted block) is assigned to the free SH stretches of the ion core. The frequency of this band is very close to the free SH stretch band (2558 cm^–1^) of the H_3_S^+^ ion core in H^+^(H_2_S)_*n*_ observed in our recent study.^21^ The two relatively higher frequency bands at around 2595 and 2610 cm^–1^ (the bands in the blue and green dotted blocks) are assigned to the symmetric (*ν*_1_) and asymmetric (*ν*_3_) SH stretching bands of the neutral H_2_S moiety, respectively, which is solvating the ion core as an H-bond acceptor. These band frequencies are also very close to the corresponding free SH bands in H^+^(H_2_S)_*n*_.^21^ The *ν*_1_ and *ν*_3_ frequencies of the neutral H_2_S monomer have been reported to be 2614 and 2628 cm^–1^, respectively.^22^ The most striking feature in the spectra is the free SH stretch band of the ion core highlighted by the red dotted block. Since the acidity of the SH bond in the ion core is enhanced with charge, the SH in the ion core is expected to be preferentially solvated (H-bonded) by neutral H_2_S in the cluster. Therefore, the free SH band of the ion core should disappear when the number of neutral H_2_S molecules in the cluster is enough to solvate all of the SH bonds of the ion core. Regardless of whether the ion core is of the hemi-bonded or proton-transferred type, the free SH of the ion core should exist in (H_2_S)_3_^+^. Assuming the ion core is the proton-transferred type, the free SH stretch in the core is supposed to disappear at *n* = 4.^9^ In the observed spectra, however, the free SH band actually disappears at *n* = 6. This clearly demonstrates that the ion core of the clusters is of the hemi-bonded type (H_2_S∴SH_2_)^+^ which has four SH bonds. The solvation of the SH bonds of the hemi-bond ion core is completed at *n* = 6. Moreover, this result indicates that the hemi-bonded ion core is stable with respect to solvation (H-bond formation) at least up to the completion of the first solvation shell.

With the solvation of the hemi-bonded ion core, the H-bonded SH stretch band of the ion core is expected to appear in the spectra. In the region below 2300 cm^–1^, a very broad absorption is seen, and this absorption is attributed to the H-bonded SH of the ion core. As the cluster size increases, weakening of the hydrogen bond of the ion core occurs as the (H_2_S)_*n*_^+^ cluster accommodates more H_2_S species, and a blue-shift of the broad absorption feature appears. However, the position of the peak is out of the reliable measurement range of our experimental setup.

To shed light on the structures of (H_2_S)_*n*_^+^ (*n* = 3–6), theoretical methods with a good balance between reliability and efficiency are required. The high reliability of the MP2/aug-cc-pVDZ level has been demonstrated for various neutral sulfur-centered hydrogen bonded systems and for H^+^(H_2_S)_*n*._^21,23^ Unrestricted wave functions for radical cations at the MP2 level tend to be contaminated by states of higher spin multiplicity. However, for (H_2_S)_2_^+^, it has been found that the unrestricted and restricted open-shell MP2 approaches, namely UMP2 and ROMP2, predict almost the same energy difference between the hemi-bonded and proton-transferred type structures, showing comparable accuracy with the results of CCSD(T), and the deviation of the spin angular moment *S* ^2^ value under UMP2 and ROMP2 is in the acceptable range.^13^ In the present work, besides the UMP2 method, a computationally cost-effective double hybrid DFT procedure, UB2PLYPD, is also employed. By including 53% HF exchange and a 27% perturbation correlation contribution, UB2PLYPD has been demonstrated to treat spin contamination well.^24^ Using these two theoretical approaches, an exhaustive conformational search generates both hemi-bonded and proton-transferred type low-lying structures on the potential energy surface. Details of the computational results are summarized in the ESI.† For all of the sizes we searched, the energy separation between the two ion core motifs is larger than 40 kJ mol^–1^ and the hemi-bonded type is the most energetically favourable of the two. Therefore, for the proton-transferred type isomers, only the most stable structure is included in the summary for *n* ≥ 4 (we should note that the structures of the proton-transferred type isomers are essentially the same as those of the corresponding H^+^(H_2_S)_*n*_, which have recently been reported by our group^21^). The predominance of the hemi-bonded type and the relative energy order are irrespective of the choice of the theoretical level, UMP2/aug-cc-pVDZ or UB2PLYPD/aug-cc-pVDZ (details are seen in the Table SI-1 in the ESI†). Essentially the same conclusion has also been reported by Do and Besley for (H_2_S)_*n*_^+^ (*n* = 2–4) by searching the isomers through the Basin–Hopping approach and subsequent structural optimization at the CCSD(T)/aug-cc-pVDZ level of theory.^12^ In our computations, the UMP2 method yields an *S* ^2^ value with a small average deviation of 0.021 compared to the exact value (0.75), and the spin contamination is not serious. Furthermore, the simulated spectra calculated using the UMP2 method show better agreement with the experimental spectra than those calculated using UB2PLYPD (details are shown in Tables SI-2 and SI-3 in ESI†). Thus, in the following, UMP2/aug-cc-pVDZ is utilized as the main theoretical method.

**Fig. 3:**
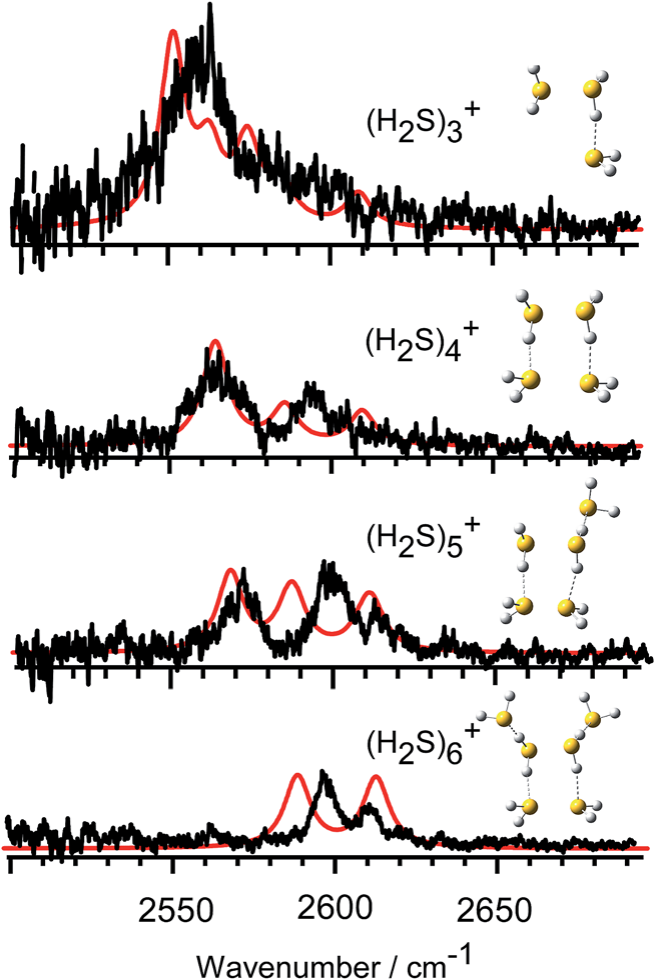
Comparison between the observed spectra and harmonic spectra of the most stable isomers of (H_2_S)_*n*_^+^ (*n* = 3–6) in the free SH region. The simulation was performed at the UMP2/aug-cc-pVDZ level of theory with a scaling factor of 0.942.

In Fig. 3, we compare the observed IR spectra of (H_2_S)_*n*_^+^ (*n* = 3–6) with the harmonic simulated spectra of the most stable isomers, which have the hemi-bonded ion core type (detailed comparison including higher energy isomers is provided in Fig. SI-1 to SI-4 in the ESI†). The frequencies of the harmonic spectra are convoluted with a Lorentzian function of 10 cm^–1^ FWHM, using a scaling factor of 0.942. The simulations reproduce well the observed spectra, supporting the qualitative assignments provided above. For the *ν*_3_ band, a non-negligible discrepancy between the observed spectra and simulations is found. The observed *ν*_3_ band intensity, relative to *ν*_1,_ seems to be remarkably suppressed. The similar suppression of the *ν*_3_ band has been also reported in H^+^(H_2_S)_*n*_ and many water analogues. This has been ascribed to differences in the internal rotation structure, dissociation yield, and transition intensity enhancement between the *ν*_1_ and *ν*_3_ bands.^21,25–27^

**Fig. 4:**
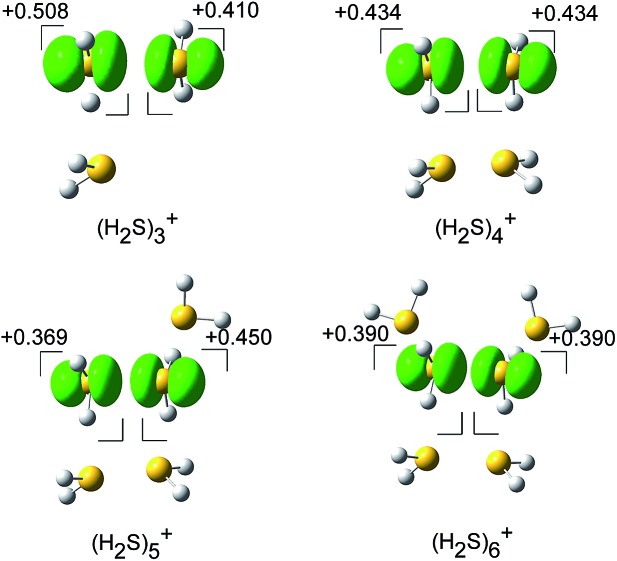
The spin density for (H_2_S)_*n*_^+^ (*n* = 3–6) (isovalue = 0.006), and the natural population analysis (NPA) charge distribution for the molecular component.

The spin densities of the most stable structures are shown in Fig. 4. The spin density (unpaired electron) is almost equally delocalized over the two H_2_S molecules, indicating the 2c–3e bond nature of the ion core. Upon solvation of the ion core, the positive charge gradually delocalizes over the solvent H_2_S moiety. Even after the completion of the first solvation shell at *n* = 6, however, the natural charge in the (H_2_S∴SH_2_)^+^ ion core is predominant, and this demonstrates the stability of the hemi-bond with respect to solvation (H-bond formation). The influence of the charge is also seen in the dissociation energy (*D*_0_), calculated using the basis set superposition error (BSSE) and zero point energy (ZPE) corrections. *D*_0_ is estimated to be 32.1, 30.2, 24.6, and 22.6 kJ mol^–1^ for *n* = 3 to 6, respectively. The gradual decrease reflects the charge delocalization of the ion core to the solvent H_2_S molecules. The *D*_0_ values of (H_2_S)_*n*_^+^ are lower than those of H^+^(H_2_S)_*n*_, wherein *D*_0_ in the first H-bonded solvation shell is 42.3 kJ mol^–1^ at the same level of theory.^21^ This is rationalized by the fact that the charge in H^+^(H_2_S)_*n*_ is primarily distributed over the single molecule of the Eigen type core H_3_S^+^ while the charge in (H_2_S)_*n*_^+^ is shared by the two H_2_S molecules of the hemi-bonded ion core.

## Experimental

(H_2_S)_*n*_^+^ clusters were generated by discharge from a supersonic jet expansion of an H_2_S/Ar gaseous mixture. Generated ions were introduced into a tandem type quadrupole mass spectrometer, wherein the cluster size of interest was selected by the first mass spectrometer (the mass resolution was set to be higher than Δ*m*/*z* = 1, and the contribution of H^+^(H_2_S)_*n*_ protonated clusters was carefully removed). Then the size-selected clusters were introduced into an octopole ion guide, therein, the clusters were irradiated by the tuneable IR light from an OPO/OPA system (LaserVision) pumped by an Nd-YAG laser (Continuum PL-8000), and the fragment ions were monitored by the second quadrupole mass spectrometer. IR spectra were recorded by monitoring the fragments in the single H_2_S loss channel while scanning the IR frequency in the 2300–2700 cm^–1^ region. The observed spectra were normalized by the IR light power and the band frequencies were calibrated by the absorption lines of CO_2_ and CH_4_. All calculations were conducted using the Gaussian09 program package.^28^

## Conclusion

In summary, through an IR spectroscopic study of the SH stretch region, we have experimentally proven the hemi-bonded motif of the ion core in (H_2_S)_*n*_^+^ (*n* = 3–6) which has been predicted by the theoretical calculations. In (H_2_S)_*n*_^+^, the hemi-bonded ion core motif is much more stable than the proton transferred ion core motif. The hemi-bonded ion core motif is stable towards solvation at least up to the completion of the first solvation shell.
