# Correction: Spectroscopic observation of two-center three-electron bonded (hemi-bonded) structures of (H_2_S)_*n*_^+^ clusters in the gas phase

**DOI:** 10.1039/c8sc90072h

**Published:** 2018-04-03

**Authors:** Dandan Wang, Asuka Fujii

**Affiliations:** a Department of Chemistry , Graduate School of Science , Tohoku University , Sendai 980-8578 , Japan . Email: asukafujii@m.tohoku.ac.jp

## Abstract

Correction for ‘Spectroscopic observation of two-center three-electron bonded (hemi-bonded) structures of (H_2_S)_*n*_^+^ clusters in the gas phase’ by Dandan Wang *et al.*, *Chem. Sci.*, 2017, **8**, 2667–2670.



## 

The authors regret that some important references were omitted from the original article. These references are presented herein.

The experimental observation of the sulfur–sulfur hemi-bond was pioneered by Asmus and coworkers.^1^–^6^ They observed transient absorption due to the σ*–σ electronic transition in solution. Moreover, they observed the transient absorptions of the hemi-bonds of sulfa with a variety of counter atoms as well as those of N–N and I–I hemi-bonds.^7^–^11^ The electronic spectrum of (H_2_S)_2_^+^ in aqueous solution was also reported by Asmus, though detailed structural information is difficult to extract from the broadened electronic transition.^3^

The S–S hemi-bond in gas phase molecules was first reported using mass spectrometry.^5^ The stable dimer cation formation of bis(isopropyl)sulfide was observed and hemi-bond formation was proposed on the basis of the fragmentation pattern. Gas phase dimerization equilibrium measurement of dimethyl sulfide cations has also suggested formation of the S–S hemi-bond.^12^ Very recently, infrared Stark spectroscopy was applied to Cl–NH_3_ in He droplets,^13^ and hemi-bond formation was concluded by the shift of the NH stretch and dipole moment measurements. This result is consistent with the prediction by high level computation of similar systems.^14^

Theoretical calculations of the S–S hemi-bond were first performed by Clark for (H_2_S)_2_^+^,^15^ and the series of his study has been extended to a variety of hemi-bonded systems.^16^–^18^

The Royal Society of Chemistry apologises for these errors and any consequent inconvenience to authors and readers.
