# Supplementary material for: Spectroscopic observation of two-center three-electron bonded (hemi-bonded) structures of (H2S)n + clusters in the gas phase
Source: Chem Sci. 2017 Jan 11;8(4):2667–70. doi: 10.1039/c6sc05361k (PMC5433515; doi:10.1039/c6sc05361k)
Supplement: Supplementary file 1 [file SC-008-C6SC05361K-s001.pdf]

Electronic Supplementary Material (ESI) for Chemical Science.
This journal is © The Royal Society of Chemistry 2017

Electronic Supplementary Information of

Spectroscopic Observation of Two-Center Three-Electron Bonded (Hemi-Bonded) Structures of the $(H_2S)_n^+$ Clusters in the Gas Phase

Dandan Wang, Asuka Fujii*

Department of Chemistry, Graduate School of Science

Tohoku University, Sendai 980-8578, Japan

*Email asukafujii@m.tohoku.ac.jp

Contents

Table S-1 Calculated relative energies of the stable isomers of $(H_2S)_n^+$ (n = 3 - 6).

Table SI-2 Calculated harmonic frequencies of the isomers of $(H_2S)_4$ at the UMP2/aug-cc-pVDZ level.

Table SI-3 Calculated harmonic frequencies of the isomers of $(H_2S)_4^+$ at the UB2PLPYD/aug-cc-pVDZ level.

Figure SI-1 Comparison between the observed and simulated spectra of $(H_2S)_3^+$.

Figure SI-2 Comparison between the observed and simulated spectra of $(H_2S)_4^+$.

Figure SI-3 Comparison between the observed and simulated spectra of $(H_2S)_5^+$.

Figure SI-4 Comparison between the observed and simulated spectra of $(H_2S)_6^+$.

Table S1-1. Calculated relative energies in kJ/mol of the isomers of $(H_2S)_n^+$ (n = 3 - 6) at the UMP2/aug-cc-pVDZ and UB2PLYPD/aug-cc-pVDZ levels. Spin angular moment $<S^2>$ values are also shown.

Structure[a]	UMP2/aug-cc-pVDZ	$<S^2>$	UB2LYPD/aug-cc-pVDZ	$<S^2>$
**3-1**	0	0.7776	0	0.7609
**3-2**	41.6	0.7619	48.4	0.7560
**3-3**	44.7	0.7637	50.9	0.7569
**3-4**	54.6	0.7611	92.2	0.7557
**3-5**	80.6	0.7611	63.4	0.7556
**4-1**	0	0.7778	0	0.7610
**4-2**	4.63	0.778	0.840	0.7612
**4-3**	6.01	0.7759	2.76	0.7607
**4-4**	9.13	0.7773	N/A	N/A
**4-5**	42.8	0.7616	46.6	0.7559
**5-1**	0	0.7775	0	0.7609
**5-2**	3.28	0.7776	5.85	0.7610
**5-3**	10.7	0.7623	N/A	N/A
**5-4**	4.76	0.7768	8.86	0.7608
**5-5**	46.0	0.7611	53.4	0.7556
**6-1**	0	0.7780	0	0.7610
**6-2**	2.87	0.7774	N/A	N/A
**6-3**	6.01	0.7770	6.74	0.7608
**6-4**	46.6	0.7611	57.1	0.7556

[a] The label of the structure corresponds to those in the following Tables and Figures.

Table SI-2. Calculated harmonic frequencies of the isomers of $(H_2S)_4^+$ at the UMP2/aug-cc-pVDZ level. All units are in cm^{-1}. The hemi-bonded type isomers are scaled by 0.942 and the proton-transferred type isomer is scaled by 0.946. The corresponding experimental values are listed for comparison.

	**Exp.**	**4-1**	**4-2**	**4-3**	**4-4**	**4-5**[a]
stretch of H-bonded SH in the ion core		2210<br>2243	2166<br>2182	2232<br>2266	2150	1949<br>2021<br>2138
stretch of SH• radical						2584
stretch of free SH in the hemi-bonded core	<u>2565</u>	2565<br>2566	2565<br>2567	2555<br>2578	2548<br>2564<br>2585[b]	
sym. stretch of free SH in H-bonded H_2S	<u>2593</u>	2586<br>2587	2585[c]<br>2585[c]	2586<br>2588	2585[b]	2592<br>2593
sym. stretch of free SH in charge-dipole bound H_2S					2593	
asym. stretch of free SH in H-bonded H_2S	<u>2610</u>	2610<br>2611	2609[c]<br>2609[c]	2610<br>2612	2610	2615<br>2617
asym. stretch of free SH in charge-dipole bound H_2S					2618	

[a] Proton-transferred type.

[b] These two vibrational modes are heavily mixed.

[c] Degenerated frequencies.

Table SI-3. Calculated harmonic frequencies of the isomers for $(H_2S)_4^+$ at the UB2PLPYD/aug-cc-pVDZ level. All units are in cm^{-1}. The hemi-bonded type isomers are scaled by 0.9607 and the proton-transferred type isomer is scaled by 0.963. The corresponding experimental values are listed for comparison.

	**Exp.**	**4-1**	**4-2**	**4-3**	**4-5**[a]
stretch of H-bonded SH in the ion core		2144<br>2195	2094<br>2126	2161<br>2194	1884<br>1963<br>2107
stretch of SH• radical					2580
stretch of Free SH in the hemi-bonded core	<u>2565</u>	2576<br>2577	2574<br>2576	2568<br>2582	
sym. stretch of H-bonded H_2S	<u>2593</u>	2593[b]<br>2593[b]	2587[b]<br>2587[b]	2590<br>2591	2593<br>2594
asym. stretch of H-bonded H_2S		2608[b]<br>2608[b]	2603[b]<br>2603[b]	2606[b]<br>2606[b]	2608<br>2609

[a] Proton-transferred type

[b] Degenerated frequencies

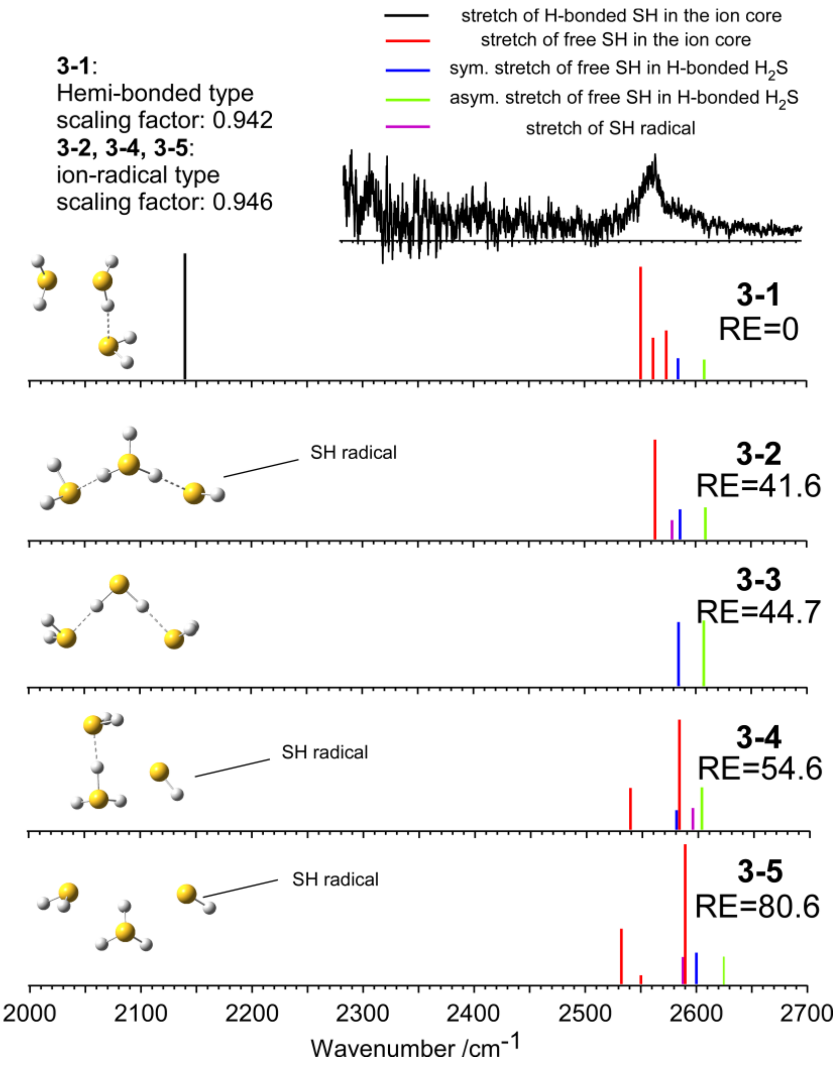

Figure SI-1. Comparison between the observed and simulated spectra of $(H_2S)_3^+$. Colors of the sticks represent types of stretch modes, and they are shown on the top of the panel. Relative energy (RE) is also shown in kJ/mol. Since the intensity of the stretch of H-bonded SH in the ion core is about 100~400 times greater than those of the free SH stretches in the neutral H_2S moiety, its stick is simply cut for a clear presentation. The stretch frequencies of H-bonded SH in the ion core of **3-2**, **3-3** and **3-4** are out of the displayed range; 1690 and 1945 cm^{-1} for **3-2,** 1573 and 1754 cm^{-1} for **3-3**, and 1368 cm^{-1} for **3-4.**

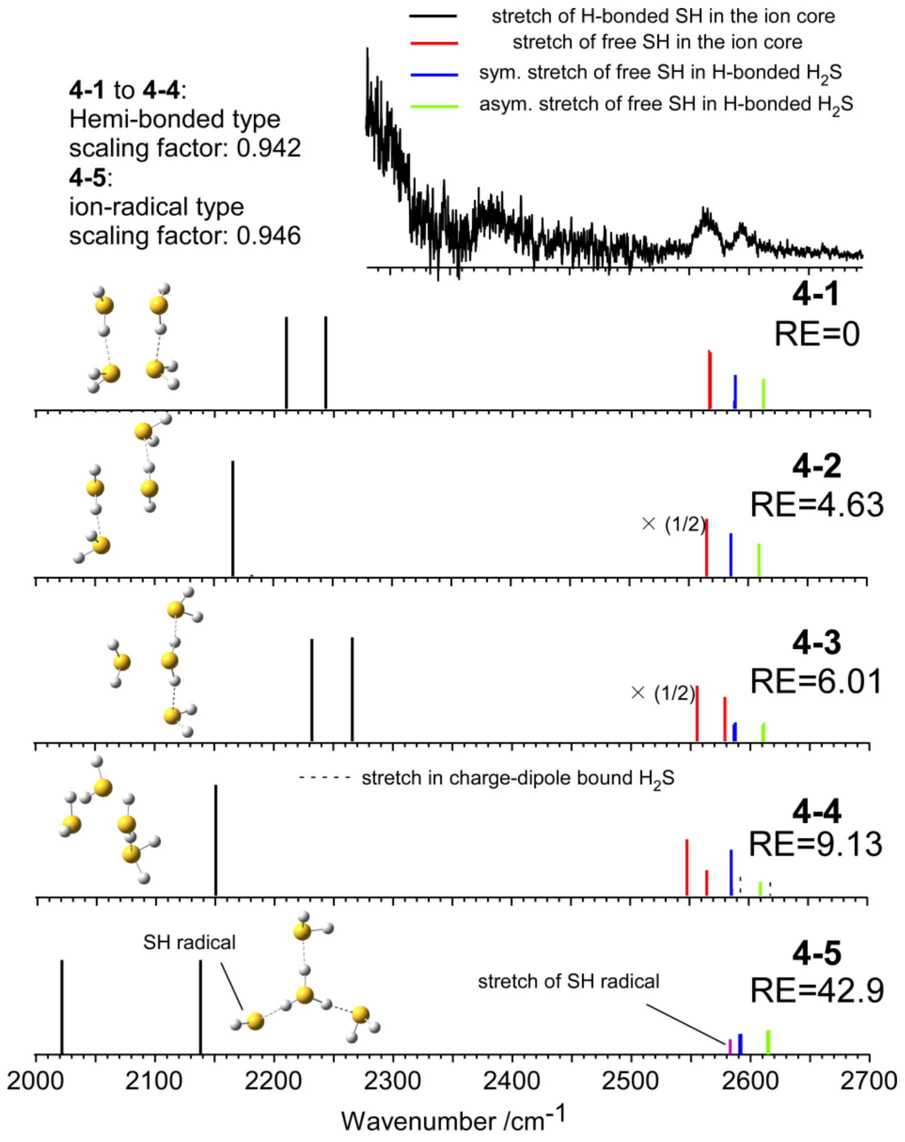

Figure SI-2. Comparison between the observed and the simulated spectra for $(H_2S)_4^{+}$. Colors of the sticks represent types of stretch modes, and they are shown on the top of the panel. Relative energy (RE) is also shown in kJ/mol. Since the intensities of the stretches of H-bonded SH in the ion core are about 100~400 times greater than that of the free SH stretches in the neutral H_2S moiety, their sticks are simply cut for a clear presentation. The two bands of stretches of H-bonded SH in the hemi-bonded core of **4-2** are degenerated. Another stretch of H-bonded SH in the ion core of **4-5** is located at 1949 cm^{-1}. For **4-4**, one H_2S is bound to the hemi-bonded ion core by the charge-dipole interaction.

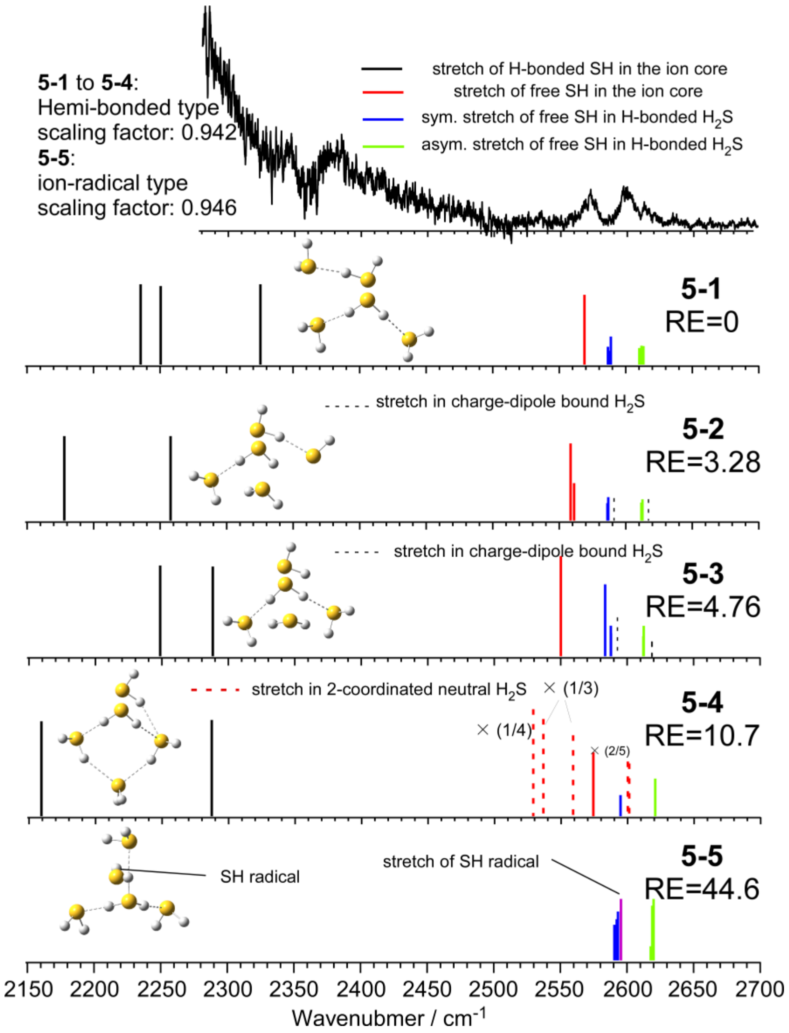

Figure SI-3. Comparison between the observed and the simulated spectra for $(H_2S)_5^+$. Colors of the sticks represent types of stretch modes, and they are shown on the top of the panel. Relative energy (RE) is also shown in kJ/mol. Since the intensities of the stretches of H-bonded SH in the ion core are about 100~400 times greater than that of the free SH stretches in the neutral H_2S moiety, their sticks are simply cut for a clear presentation. In **5-2** and **5-3**, there are the stretch vibrational modes of charge-dipole bound H_2S, which are denoted by black dotted lines. In **5-4**, the SH stretches arising from the 2-coordinated H_2S sites are denoted by red dotted lines. The stretches of H-bonded SH in the ion core of **5-5** locate at 1889, 2011, and 2106 cm^{-1}.

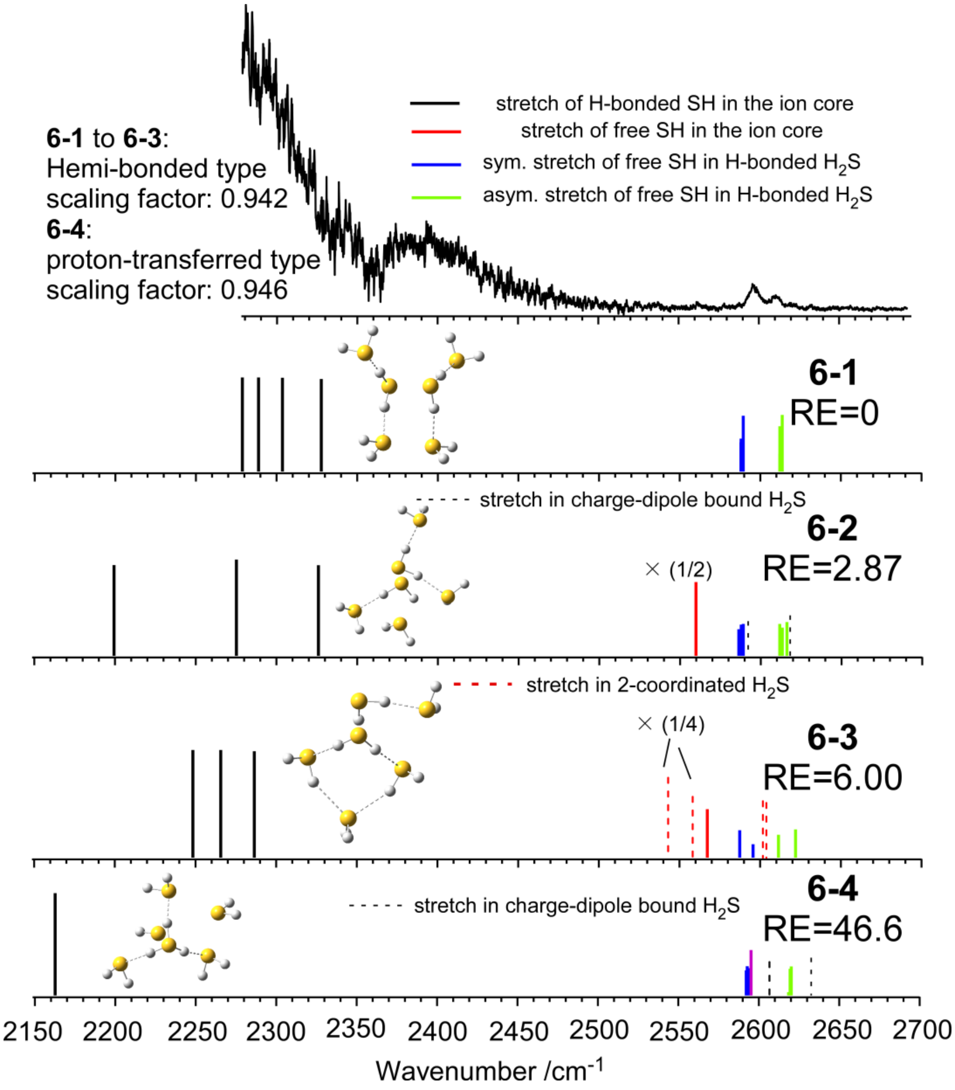

Figure SI-4. Comparison between the observed and the simulated spectra for $(H_2S)_6^+$. Colors of the sticks represent types of stretch modes, and they are shown on the top of the panel. Relative energy (RE) is also shown in kJ/mol. Since the intensities of the stretches of H-bonded SH in the ion core are about 100~400 times greater than that of the free SH stretches in the neutral H_2S moiety, their sticks are simply cut for a clear presentation. For **6-2** and **6-4**, there are the stretch vibrational modes of charge-dipole bound H_2S, which are denoted by black dotted lines. The stretches arising from the 2-coordinated H_2S sites in **6-3** are denoted by red dotted lines. Another two stretches of H-bonded SH in the ion core of **6-4** locate at 1897 and 1995 cm^{-1}.
